# Impact of dietary arginine supplementation on immune responses and growth performance in Newcastle disease virus‐infected broiler chicks

**DOI:** 10.1002/vms3.1571

**Published:** 2024-08-07

**Authors:** Fatemeh Izadi Yazdanabadi, Gholamali Moghaddam, Mohsen Akbari, Mehdi Abbasabadi

**Affiliations:** ^1^ Department of Animal Science, Faculty of Agriculture University of Tabriz Tabriz Iran; ^2^ Department of Animal Science, College of Agriculture and Natural Resources Razi University Kermanshah Iran; ^3^ Department of Animal Science, Faculty of Agriculture University of Birjand Birjand Iran

**Keywords:** broiler chicks, growth, immunity, inflammation, Newcastle

## Abstract

**Background:**

Newcastle disease (ND) poses significant challenges within the poultry industry, leading to increased mortality rates, compromised growth, weakened immunity and elevated levels of inflammation.

**Objective:**

This study explores the potential of dietary arginine supplementation to ameliorate these adverse effects of ND, leveraging arginine's well‐documented benefits in enhancing growth and immune responses.

**Methods:**

A total of 480 one‐day‐old male broiler chicks were meticulously categorised into eight groups, encompassing both infected and noninfected cohorts. These chicks received diets with arginine levels at 85%, 100%, 125% and 150% of recommended standards. The study entailed a comprehensive examination of clinical manifestations, growth performance metrics, haemagglutination inhibition (HI) test results, and serum concentrations of proinflammatory cytokines, adrenocorticotropic hormone (ACTH), and cortisol (CORT).

**Results:**

The infection significantly curtailed feed consumption (*p* = 0.0001) and weight gain (*p* = 0.0001) while concurrently depressing HI titres. Additionally, infected chicks experienced an exacerbated feed conversion ratio (*p* = 0.0001), escalated mortality rates (*p* = 0.0001), and elevated serum concentrations of proinflammatory cytokines (*p* = 0.0001), ACTH (*p* = 0.0001), and CORT (*p* = 0.0001). Remarkably, dietary arginine supplementation effectively mitigated the adverse impacts of ND infection on growth, immune responses and proinflammatory cytokine levels. In the context of ND infection, mortality rates and inflammation surge, while growth and immunity are significantly compromised.

**Conclusions:**

The strategic inclusion of arginine in the diet emerges as a potent strategy to counteract the deleterious effects of ND. Supplementation with arginine at levels exceeding the conventional dietary recommendations is recommended to alleviate the detrimental consequences of ND effectively.

## Graphical abstract

✓ This study investigates the potential of dietary arginine supplementation to mitigate these adverse effects of ND, capitalising on arginine's well‐established benefits in promoting growth and immune function.

✓ ND infection significantly increased serum levels of proinflammatory cytokines and reduced feed intake (*p* = 0.0001) and weight gain (*p* = 0.0001), along with a decline in HI titres.

✓ Supplementation with arginine levels surpassing conventional dietary recommendations is advisable to effectively mitigate the adverse consequences of ND.

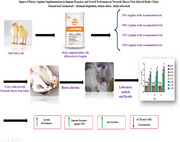

## 1 INTRODUCTION

The surge in population growth has led to an increased demand for meat consumption. Nevertheless, animal diseases have reduced productivity, with Newcastle disease (ND) being a notable example (Yu et al., 2021). Newcastle disease virus (NDV) is the causative agent behind ND, classified within the avian avulavirus family. This affliction harms the poultry industry (Fan et al., 2019), precipitating economic setbacks encompassing mortality, condemnation of slaughterhouse carcasses, suboptimal production, and escalated treatment expenditures (Jafari et al., 2019). The continuous prevalence of this disease in developing countries poses a significant threat to the poultry sector (Anjum et al., 2020).

The innate immune system is crucial in combating viral infections (Rehman et al., 2017). Research has additionally demonstrated that proinflammatory cytokines constitute a vital component of the immune response, aiding in regulating potential adverse effects and restoring homeostasis (Borghetti et al., 2009; Borghetti et al., 2011). Vaccination is pivotal for infection prevention, viral replication inhibition, and reducing prevalence and mortality rates (Jafari et al., 2019). However, the administration of vaccines induces stress and may lead to immune dysfunction (Kaab et al., 2018). Furthermore, vaccines are susceptible to degradation when exposed to inappropriate temperatures (Asl Najjari et al., 2017). Implementing specific feeding strategies can mitigate stress and enhance immune responses. Feed additives exert control over pathogenic microorganisms while promoting the proliferation of beneficial microorganisms within the intestinal system (Vahdatpour et al., 2011).

Amino acids are pivotal in various physiological processes, including growth, immune function, antioxidant mechanisms, cell signalling, and gene expression (Castro & Kim, 2020). Arginine, an essential amino acid for avian species, is a precursor for synthesising proteins and other metabolically significant molecules, such as nitric oxide (NO) (Khajali & Wideman, 2010). Scientific studies have suggested the utilisation of arginine to ameliorate the detrimental effects of stress and diseases (Atakisi et al., 2009). Incorporating arginine into the diet has been shown to alleviate immune responses by reducing the levels of inflammatory cytokines (Guo et al., 2015). Notably, research conducted by Izadi Yazdanabadi et al. (2020a) revealed that dietary supplementation of arginine led to a decrease in serum concentrations of inflammatory cytokines and improved the growth performance of broiler chicks infected with coccidiosis.

Furthermore, positive effects of dietary arginine inclusion on the cecum microbial population and antioxidant status in both healthy and coccidia‐challenged broiler chicks were reported (Izadi et al., 2020). Recent studies have also highlighted the positive influence of arginine on enhancing intestinal morphology in broiler chicks facing coccidiosis challenges (Yazdanabadi et al., 2020b). Additionally, arginine has positively alleviated respiratory diseases (Ahmadipour et al., 2018; Wideman et al., 1995).

While ND exerts adverse effects on growth performance and immune responses, the administration of arginine may mitigate these negative consequences on growth and immunity. Nonetheless, the specific impact of arginine supplementation on improved growth and immunity in the context of NDV infection remains unexplored. Therefore, this study aims to investigate the effects of dietary arginine supplementation on growth performance, immune responses and inflammatory factors in broiler chicks afflicted with ND.

## 2 MATERIALS AND METHODS

### 2.1 Broiler chicks and grouping

The present investigation was conducted following the guidelines approved by the Animal Care Committee of (Approval No. TU 12081/December 2019). Four hundred eighty male 1‐day‐old broiler chicks of the Ross 308 strain, with an initial weight of 41±3 grams, were procured from a local hatchery. The broiler chicks were subjected to a lighting regimen consisting of 23 h of light followed by 1 h of darkness, and they were housed in pens furnished with fresh wood shavings (1 × 1 m^2^). The These broiler chicks were provided ad libitum access to water and feed, with their diets formulated based on a corn‐soybean composition. The dietary formulations adhered to the specifications outlined in the Ross catalogue (Aviagen, 2014) (Table 1) and were designed to cater to the nutritional needs of the birds during three distinct phases: the starter phase (1–10 days), the grower phase (11–24 days) and the finisher phase (25–42 days).

**TABLE 1:** Results for ACTH (pg/mL) and CORT (pg/mL) in broiler chicks.

Groups	ACTH	CORT
H85	25.10 ± 3.21^e^	1.20 ± 0.11^e^
H100	26.70 ± 2.89^e^	1.30 ± 0.12^e^
H125	25.90 ± 2.51^e^	1.32 ± 0.21^e^
H150	24.90 ± 2.51^e^	1.25 ± 0.07^e^
C85	58.20 ± 3.41^a^	4.30 ± 0.11^a^
C100	51.23 ± 2.14^b^	3.90 ± 0.10^b^
C125	43.21 ± 3.21^c^	3.50 ± 0.21^c^
C150	31.15 ± 3.21^d^	3.10 ± 0.32^d^
*p*‐value	0.001	0.001

*Note*: Different superscripts indicate significant differences between groups.

For this study, the broiler chicks were segregated into eight distinct groups, with each group containing six replications and each replication consisting of 10 broiler chicks. These groups were provided with diets supplemented with varying levels of digestible arginine, precisely at 85%, 100%, 125% and 150% of the recommended levels. The 85% and 100% levels were designated suboptimal and standard, respectively.

Infection induction was carried out on the 21st day of the rearing period. Infected and noninfected groups were maintained in separate chambers from the study's outset to prevent disease transmission. The rearing conditions and equipment were standardised across both chambers.

The experimental groups encompassed the following:

1. NDV‐infected broiler chicks fed with 85% arginine (C85).
2. NDV‐infected broiler chicks fed with 100% arginine (C100).
3. NDV‐infected broiler chicks fed with 125% arginine (C125).
4. NDV‐infected broiler chicks fed with 150% arginine (C150).
5. Noninfected broiler chicks fed with 85% arginine (H85).
6. Noninfected broiler chicks fed with 100% arginine (H100).
7. Noninfected broiler chicks fed with 125% arginine (H125).
8. Noninfected broiler chicks fed with 150% arginine (H150).

To determine the arginine content in the feed samples, an ion‐exchange High‐Performance Liquid Chromatography (HPLC) method was employed, and the resulting data are presented in Table 2.

**TABLE 2:** Digestible arginine (%) in different periods.

Period	85 % Arg	100 % Arg	125 % Arg	150 % Arg
Stater	1.164	1.37	1.172	2.055
Grower	1.045	1.23	1.537	1.845
Finisher	0.935	1.1	1.375	1.65

## 3 EXPERIMENTAL ANIMAL

### 3.1 Virus

Blood samples were collected from the wing vein of chicks on the 20th day to ensure the absence of antibodies against ND. The NDV was isolated from infected chicks in the field. The procedures for virus amplification, stock preparation, and dilution followed the methodology described by Younus et al. (2018). The isolated pathotype was confirmed as virulent, exhibiting a mean death time of 52 h and an intracerebral pathogenicity index of 1.83, following standard protocols and procedures recommended by others (Younus et al., 2018).

### 3.2 Disease reproduction

Each bird was infected through intranasal administration of 0.05 mL (right nasal) and intraocular injection of 0.05 mL with the NDV isolate.

### 3.3 Clinical signs and sampling

Throughout the 42‐day study period, the broiler chicks were subject to daily monitoring for clinical signs. All pathological manifestations were carefully observed, focusing on respiratory system indicators. Clinical signs were scored as per the criteria outlined by Jirjis et al. (2004). Additionally, other signs such as green diarrhoea, wing and leg paralysis, and twisted neck were monitored and scored on a scale of 0 (none) to 4 (very severe).

### 3.4 Haemagglutination inhibition (HI) test

Blood samples were collected weekly from the wing vein, subjected to centrifugation for 12 min at 1300 × *g*, and evaluated for antibody responses. The obtained serums from each broiler chick were stored at −70°C until further analysis. HI responses were assessed following the procedure described by Asl Najjari et al. (2017).

### 3.5 Measurement of inflammatory responses

At 42 days of age, blood samples were collected from two birds per replication via the wing vein and centrifuged for 12 min at 1300 × *g* and 4°C. Serum NO concentration was determined using a specified commercial kit (NO: ZELLbio, Germany; Cat No.: ZB‐NO.96 A) according to the manufacturer's instructions and measured using a spectrophotometer.

The serum concentrations of proinflammatory cytokines, including IL‐1β (MBS2510251; MyBioSource Company), IL‐2 (ab273263; Abcam Company), IL‐6 (MBS268769, MyBioSource Company), IFN‐γ (MBS778165; MyBioSource Company), and TNF‐α (MBS2509660; MyBioSource), were assessed using ELISA kits following the producer Companies' instructions. Additionally, the concentrations of serum adrenocorticotropic hormone (ACTH) and cortisol (CORT) were determined using commercial kits from Beijing North Institute of Biological Technology, Beijing, China, as recommended by the manufacturer.

### 3.6 Growth performance

At the end of the study period, the broiler chicks were harvested and weighed after a 3‐h fasting period. Performance parameters such as average daily gain (ADG), average daily feed intake (ADFI), and feed conversion ratio (FCR) were calculated. Mortality data were recorded daily.

## 4 DATA ANALYSIS

This study employed a completely randomised design with a factorial arrangement involving the factors of infection (NDV‐infected and noninfected) and dietary supplementation with arginine (at levels of 85%, 100%, 125% and 150% of recommended levels). The normality and nonnormal data were assessed using the Kruskal–Wallis test. Data analysis was conducted using SPSS software (Version 23), and graphical representations were generated using Graph Pad Prism software (Version 6.07).

## 5 ETHICAL STANDARDS

All the principles for care and treatment of broiler chicks were confirmed by the Ethical Committee of Tabriz University (TU, 110254, June 2015).

## 6 RESULTS

### 6.1 Clinical findings score

**FIGURE 1:**
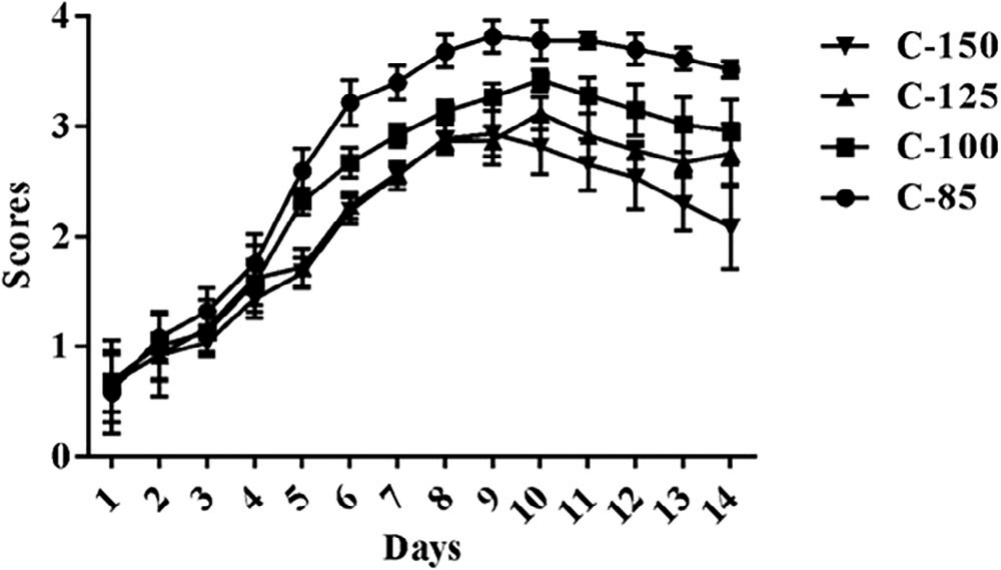
Clinical scores in NDV‐infected broiler chicks. C85–C150 represents broiler chicks afflicted with Newcastle disease and receiving diets supplemented with 85%, 100%, 125% and 150% arginine.

Figure 1 presents the outcomes of clinical scoring following Newcastle disease infection. Initially, there were no significant discrepancies in scores among the various groups during the first 4 days postinfection. However, broiler chicks with arginine at 100%, 125% and 150% exhibited diminished scores from days 5 to 14 following infection induction. Notably, the lowest scores were recorded in broiler chicks receiving a 150% arginine supplementation. Clinical manifestations reached their zenith on day 9 postinfection, with consistent signs observed in the C85 group. The highest incidences of paralysis and green diarrhoea were documented in the C85 group, while the lowest occurrences were noted in the C150 group.

### 6.2 Mortality

**FIGURE 2:**
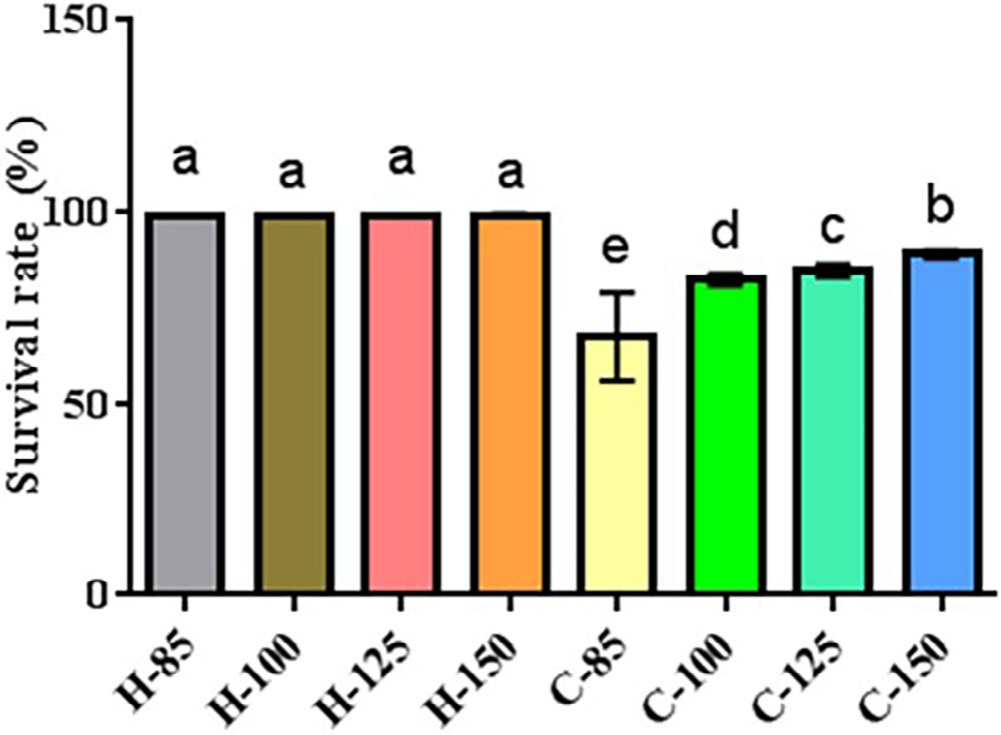
Mortality results of broiler chicks. Dissimilar letters (a–e) indicate significant differences among groups. C85–C150 represents broiler chicks afflicted with Newcastle disease and receiving diets supplemented with 85%, 100%, 125% and 150% arginine. H85–H150 represents noninfected broiler chicks receiving diets supplemented with 85%, 100%, 125% and 150% arginine.

Figure 2 displays the mortality outcomes. The Newcastle disease infection had a pronounced impact on mortality (*p* = 0.0001). NDV‐infected broiler chicks receiving arginine displayed lower mortality rates when compared to those receiving suboptimal arginine levels (85%). Noninfected broiler chicks exhibited no statistically significant differences in mortality (*p* = 0.856).

### 6.3 Inflammatory responses

**FIGURE 3:**
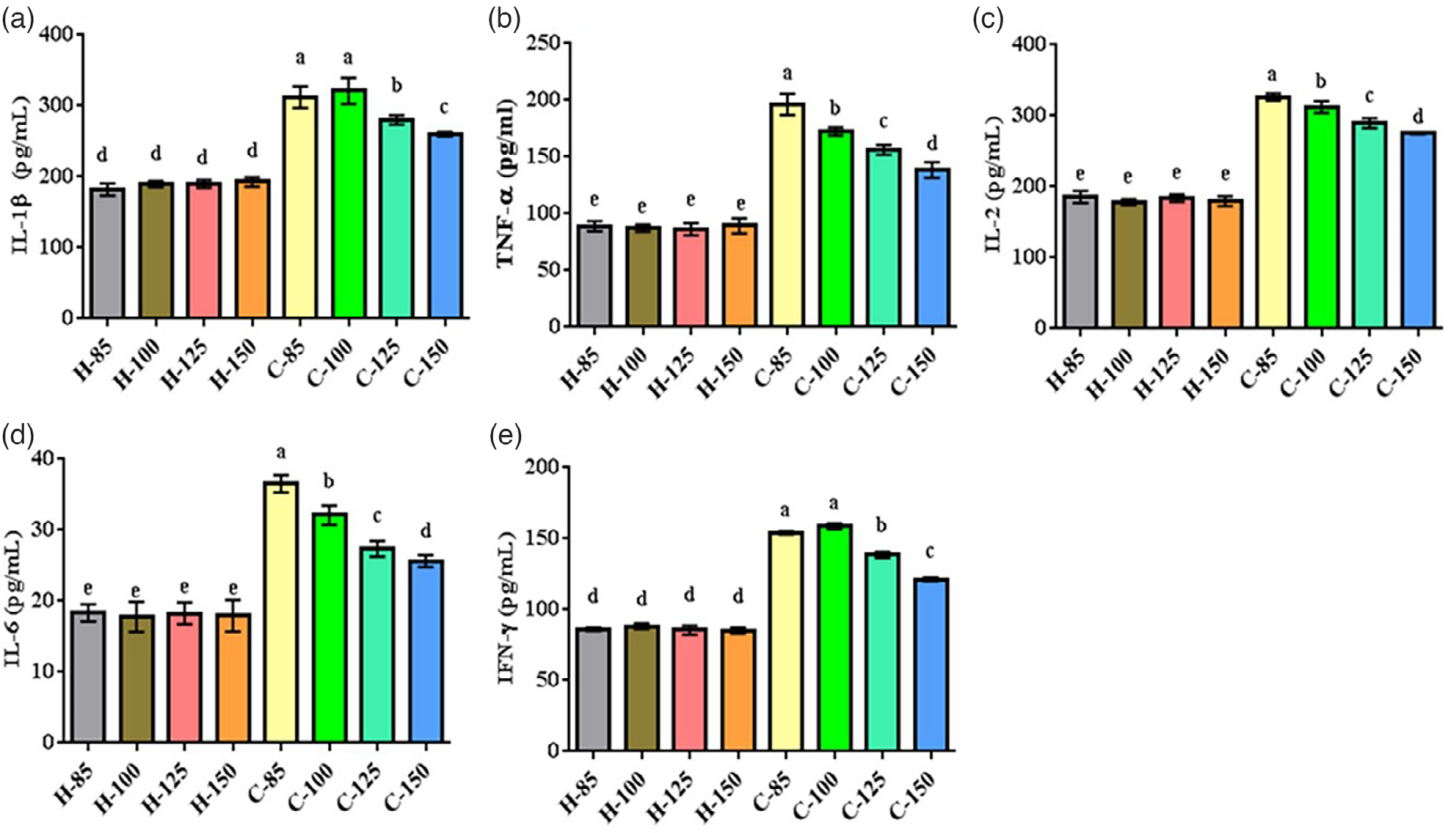
Inflammatory responses in infected and noninfected broiler chicks. Dissimilar letters (a–e) indicate significant differences among groups. C85–C150 represents broiler chicks afflicted with Newcastle disease and receiving diets supplemented with 85%, 100%, 125% and 150% arginine. H85–H150 represents noninfected broiler chicks receiving diets supplemented with 85%, 100%, 125% and 150% arginine.

Figure 3 illustrates the inflammatory responses in broiler chicks. The induction of infection led to a notable increase in serum proinflammatory cytokine concentrations (*p* = 0.0001). Conversely, noninfected broiler chicks displayed no significant variations in inflammatory responses (*p >* 0.05). Broiler chicks supplemented with 125% and 150% arginine exhibited lower proinflammatory cytokine concentrations than those receiving other supplementation levels. The lowest concentrations were observed in broiler chicks with the highest arginine level.

### 6.4 Serum nitric oxide

**FIGURE 4:**
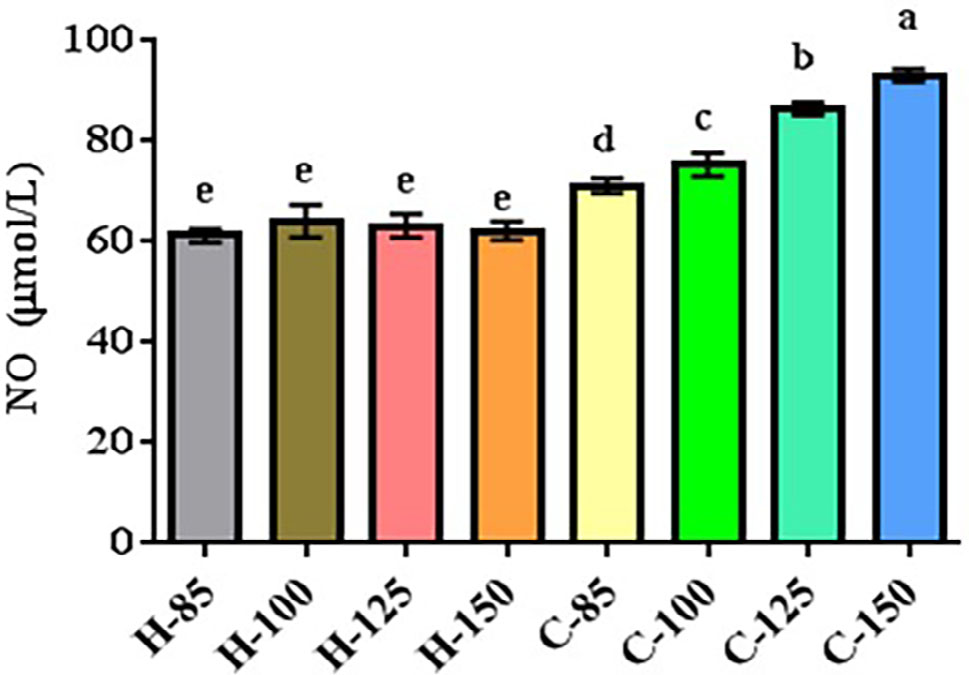
Serum concentration of nitric oxide in infected and noninfected broiler chicks. Dissimilar letters (a–e) indicate significant differences among groups. C85–C150 represents broiler chicks afflicted with Newcastle disease and receiving diets supplemented with 85%, 100%, 125% and 150% arginine. H85–H150 represents noninfected broiler chicks receiving diets supplemented with 85%, 100%, 125% and 150% arginine.

Figure 4 provides insights into serum nitric oxide concentrations. The results indicated no significant differences in noninfected broiler chicks (*p* = 0.659). Our findings demonstrate that dietary arginine supplementation increased serum nitric oxide concentrations, with the highest levels observed in broiler chicks fed diets enriched with 150% arginine.

### 6.5 Serum concentrations of ACTH and CORT

Table 1 presents the results regarding the effects of arginine supplementation on day 42. No significant differences were detected among healthy groups for ACTH (*p* = 0.718) and CORT (*p* = 0.693). However, significant disparities emerged among the Newcastle‐infected groups (*p* = 0.001). The addition of arginine led to a dose‐dependent reduction in serum ACTH and CORT concentrations.

### 6.6 Immune responses

**FIGURE 5:**
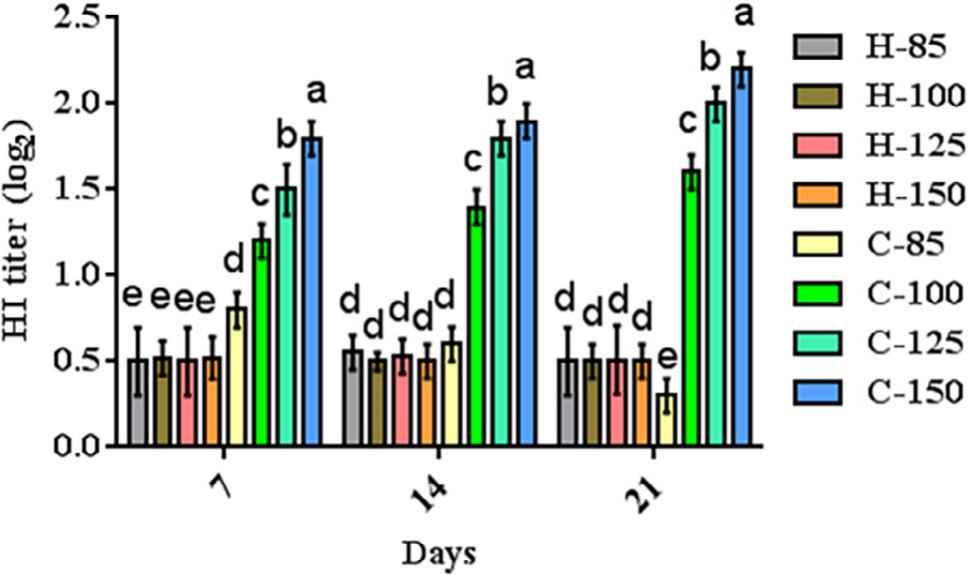
HI titre (log_2_) results in infected and noninfected broiler chicks. Dissimilar letters (a–e) indicate significant differences among groups on the same day. C85–C150 represents broiler chicks afflicted with Newcastle disease and receiving diets supplemented with 85%, 100%, 125% and 150% arginine. H85–H150 represents noninfected broiler chicks receiving diets supplemented with 85%, 100%, 125% and 150% arginine.

Figure 5 showcases the results of HI titres in broiler chicks. Arginine supplementation had no significant impact on HI titres in noninfected broiler chicks on days 7 (*p* = 0.765), 14 (*p* = 0.692), and 21 (*p* = 0.583). Notably, broiler chicks receiving higher arginine levels exhibited the highest titres compared to their counterparts (*p* = 0.0001). The lowest and highest titres were recorded in broiler chicks with 85% and 150% arginine, respectively.

### 6.7 Growth performance

**FIGURE 6:**
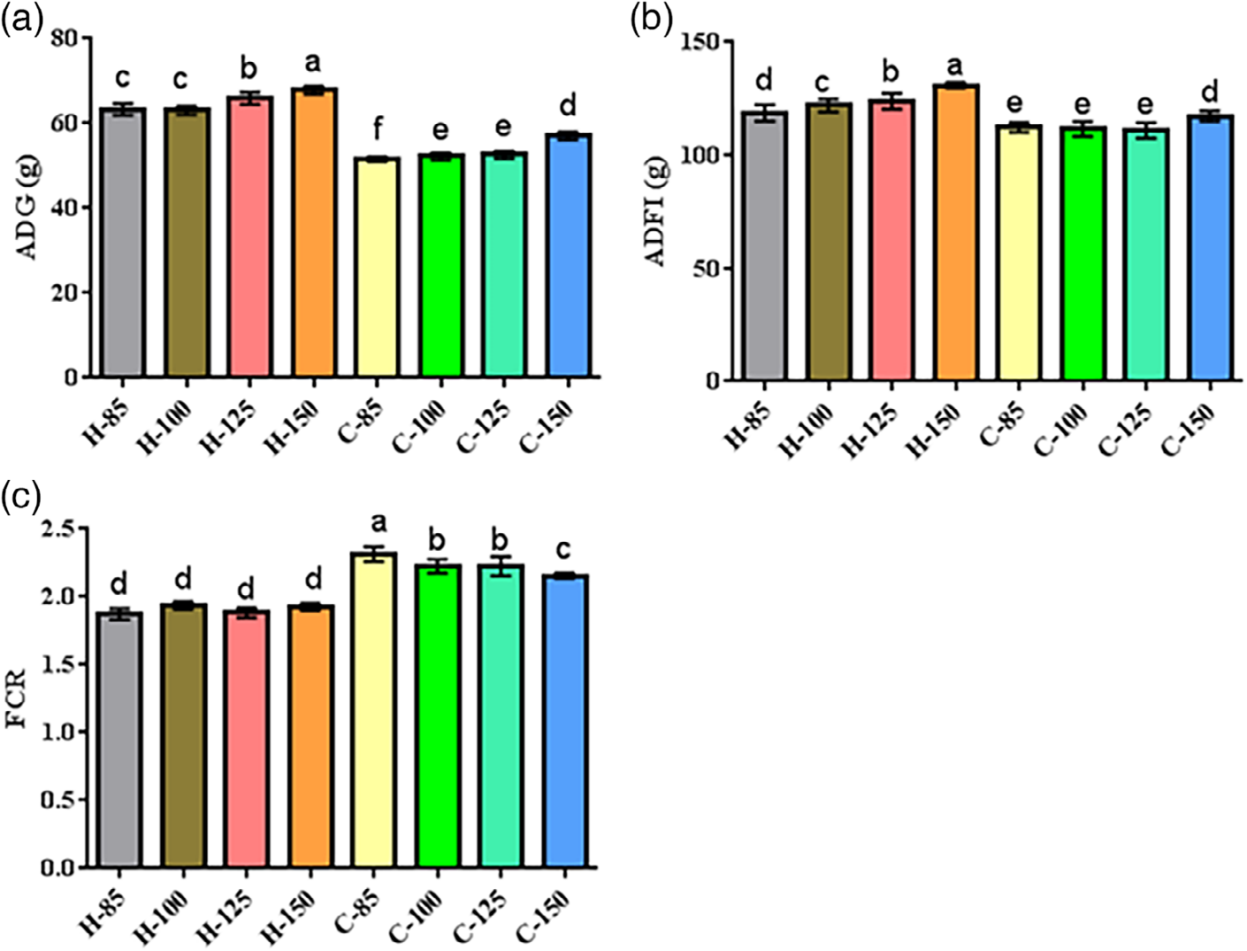
Growth performance results of broiler chicks. Dissimilar letters (a–e) indicate significant differences among groups. C85–C150 represents broiler chicks afflicted with Newcastle disease and receiving diets supplemented with 85%, 100%, 125% and 150% arginine. H85–H150 represents noninfected broiler chicks receiving diets supplemented with 85%, 100%, 125% and 150% arginine.

Figure 6 illustrates the growth performance outcomes in both NDV‐infected and noninfected broiler chicks. Newcastle disease infection significantly influenced growth performance (*p* = 0.0001) and mortality (*p* = 0.0001). Noninfected broiler chicks displayed the highest ADG and ADFI and the lowest FCR and mortality rates. Arginine supplementation also yielded significant effects on growth performance (*p* = 0.0001). Noninfected broiler chicks receiving higher arginine levels (125% and 150%) demonstrated superior ADG and ADFI compared to other groups. Furthermore, NDV‐infected broiler chicks fed with 150% arginine displayed higher ADFI and ADG than other NDV‐infected broiler chicks. Noninfected broiler chicks exhibited no significant differences in FCR (*p* = 0.693). In summary, the induction of infection adversely affected growth performance, but including arginine in the diet mitigated these negative impacts.

## 7 DISCUSSION

ND is a significant ailment within the global poultry industry (Li et al., 2020). Vaccination stands as the foremost approach to combat this affliction. Nevertheless, dietary strategies can mitigate its impact. In this present investigation, including arginine in the diet reduced clinical scores. Broiler chicks fed a suboptimal arginine level exhibited severe clinical signs and lesions, which can be attributed to pronounced inflammation. The initial days did not display significant score variations, possibly due to the virus's limited impact during this early stage. Our results revealed elevated serum concentrations of proinflammatory cytokines in broiler chicks fed lower arginine levels.

Conversely, higher arginine levels decreased cytokine levels, suggesting a direct link between inflammation and clinical scores. Similar findings were reported by Dwars et al. (2008), associating higher scores with severe inflammation. Ahmadipour et al. (2018) also noted that dietary arginine supplementation reduced respiratory symptoms through nitric oxide production. Our study indicated that arginine supplementation elevated nitric oxide levels, potentially alleviating clinical signs. Consequently, arginine supplementation mitigates clinical symptoms by reducing inflammation and increasing nitric oxide production.

The induction of infection had adverse effects on growth performance and increased mortality rates. ND primarily affects the respiratory system and alters metabolism in broiler chicks. This disorder results in reduced food consumption and weight gain. Infection disrupts metabolism, diverting a significant portion of feed toward antibody production and inflammatory responses, thus diminishing weight gain. Amino acids are not utilised for weight gain during infection. High levels of arginine supplementation improved growth performance in both noninfected and NDV‐infected broiler chicks. Several studies have reported the positive influence of arginine on growth performance under infection conditions (Tan et al., 2014; Yazdanabadi et al., 2020a).

Nevertheless, our findings demonstrated that recommended and lower arginine levels did not positively impact growth performance during Newcastle infection. For noninfected conditions, recommended arginine levels sufficed, while higher levels were advisable under nonstressful conditions. Under Newcastle infection, arginine supplementation at 1.5 times the recommended level was necessary for enhanced growth performance. The improved performance in broiler chicks supplemented with arginine can be attributed to its essential role in poultry nutrition (Perez‐Carbajal et al., 2010). It contributes to body protein structure and enhances weight (Emadi et al., 2011). Broiler chicks divert feed toward antibody production during disease exposure. Arginine supplementation may aid in antibody production due to its essential nature. Haemagglutination inhibition (HI) results revealed significantly higher titres in NDV‐infected broiler chicks supplemented with 150% arginine compared to other groups. These data suggest that feed is primarily consumed for immune responses, while the body responds to infection by producing inflammatory cytokines. Broiler chicks receiving higher arginine levels had lower serum concentrations of proinflammatory cytokines, indicating reduced feed consumption for inflammatory responses and increased weight gain. Differences among infected groups could also be attributed to variations in ACTH and CORT serum concentrations. Elevated levels of ACTH and CORT over an extended period imply chronic stress, leading to reduced growth performance and apoptotic effects in immune organs, subsequently compromising immunity and growth.

Infection led to increased mortality rates, which arginine supplementation effectively reduced, corroborating findings by Younus et al. (2018) regarding increased mortality under Newcastle infection. Newcastle affects respiratory and immune systems and disrupts nutrient metabolism, resulting in mortality. As arginine is an essential amino acid in poultry, its supplementation may indirectly bolster immune responses. Studies have reported that arginine deficiency reduces nitric oxide production by macrophages, while supplementation increases CD8+ cells (Ghamari Monavvar et al., 2020). Furthermore, macrophage nitric oxide production enhances thymus function, lymphocyte mitogenesis and overall immune responses, indirectly impacting antibody titres.

Infection induction amplified the serum concentration of proinflammatory cytokines, a typical response to infection decreased inflammation correlated with elevated HI titres and nitric oxide production. Nitric oxide is a crucial mediator in innate and acquired immunity, with its concentration increasing during infection induction (Yazdanabadi et al., 2020a). The reduced serum concentration of proinflammatory cytokines in the arginine‐supplemented group may be attributed to decreased infection severity through improved gut integrity, possibly by upregulating tight junction protein mRNA content. Nitric oxide is toxic to microorganisms, reducing their population (Allen & Fetterer, 2002). Arginine is involved in nitric oxide synthesis in macrophages, and thus, increased arginine levels result in enhanced nitric oxide production, potentially improving immune responses.

## 8 CONCLUSION

In summary, ND has been found to increase injuries to the respiratory, digestive and nervous systems, as well as induce inflammation while simultaneously decreasing growth performance and suppressing the immune system in broiler chicks. However, the adverse effects of ND can be mitigated through arginine supplementation, which has been shown to improve growth performance, reduce stress responses, enhance immunity and ameliorate the inflammatory process. Therefore, it is recommended to consider dietary arginine supplementation to enhance growth and immunity in broiler chicks facing inflammation in the poultry industry.

## AUTHOR CONTRIBUTIONS

**Fatemeh Izadi Yazdanabadi**: Conceptualisation (lead); investigation (equal); methodology (equal); writing—original draft preparation (lead); writing—review & editing (equal); resources (equal). **Gholamali Moghaddam**: Project administration (lead); supervision (lead); methodology (equal); writing—review & editing (equal). **Mohsen Akbari**: Data curation (lead); formal analysis (lead); software (lead); resources (equal). **Mehdi Abbasabadi**: Investigation (equal); resources (equal); writing—review & editing (equal).

## CONFLICT OF INTEREST STATEMENT

The authors declare no conflicts of interest.

## FUNDING

No funding was received for this research.

### PEER REVIEW

The peer review history for this article is available at https://www.webofscience.com/api/gateway/wos/peer‐review/10.1002/vms3.1571.

## ETHICS STATEMENT

The authors confirm that the ethical policies of the journal, as noted on the journal's author guidelines page, have been adhered to and the appropriate ethical review committee approval has been received. All the principles for care and treatment of broiler chicks were confirmed by the Ethical Committee of Tabriz University (TU, 110254, June 2015).

## Data Availability

The data that support the findings of this study are available from the corresponding author upon reasonable request
